# Dorsal root ganglion stimulation produces differential effects on action potential propagation across a population of biophysically distinct C-neurons

**DOI:** 10.3389/fpain.2022.1017344

**Published:** 2022-10-26

**Authors:** Robert D. Graham, Amolak S. Jhand, Scott F. Lempka

**Affiliations:** ^1^ Department of Biomedical Engineering, University of Michigan, Ann Arbor, MI, United States; ^2^ Biointerfaces Institute, University of Michigan, Ann Arbor, MI, United States; ^3^ Department of Anesthesiology, University of Michigan, Ann Arbor, MI, United States

**Keywords:** dorsal root ganglion stimulation, electric stimulation, chronic pain, neuropathic pain, implanted neurostimulators, computer simulation, dorsal root ganglia, spinal cord stimulation

## Abstract

Dorsal root ganglion stimulation (DRGS) is a neurostimulation therapy used to manage chronic pain that does not respond to conventional therapies. Unfortunately, not all patients receive sufficient pain relief from DRGS, leaving them with few other treatment options. Presently, our understanding of the mechanisms of action of DRGS is incomplete, preventing us from determining why some patients do not receive analgesia from the therapy. One hypothesis suggests that DRGS augments the filtering of action potentials (APs) at the T-junction of nociceptive C-neurons. To test this hypothesis, we utilized a computational modeling approach in which we developed a population of one thousand biophysically distinct C-neuron models which each produced electrophysiological characteristics (e.g., AP height, AP duration) reported in previous experimental studies. We used this population of model C-neurons to study how morphological and electrophysiological characteristics affected the propagation of APs through the T-junction. We found that trains of APs can propagate through the T-junction in the orthodromic direction at a higher frequency than in the antidromic direction due to the decrease in axonal diameter from the peripheral to spinal axon. Including slow outward conductances in the axonal compartments near the T-junction reduced following frequencies to ranges measured experimentally. We next used the population of C-neuron models to investigate how DRGS affected the orthodromic propagation of APs through the T-junction. Our data suggest that suprathreshold DRGS augmented the filtering of APs at the T-junction of some model C-neurons while increasing the activity of other model C-neurons. However, the stimulus pulse amplitudes required to induce activity in C-neurons (i.e., several mA) fell outside the range of stimulation pulse amplitudes used clinically (i.e., typically ≤1 mA). Furthermore, our data suggest that somatic GABA currents activated directly or indirectly by the DRGS pulse may produce diverse effects on orthodromic AP propagation in C-neurons. These data suggest DRGS may produce differential effects across a population of C-neurons and indicate that understanding how inherent biological variability affects a neuron's response to therapeutic electrical stimulation may be helpful in understanding its mechanisms of action.

## Introduction

Chronic pain is a debilitating neurological disorder that affects approximately twenty percent of the world's population (1). The prevalence of chronic pain incurs more than 600 billion dollars in healthcare costs each year and contributes to the ongoing opioid epidemic (2, 3). Dorsal root ganglion stimulation (DRGS) is a non-addictive, reversible treatment for managing pain that is refractory to conventional medical management. DRGS is achieved by implanting a small electrode array in the intraforaminal space on the dorsal side of a dorsal root ganglion (DRG) (4). The electrode array applies brief electrical impulses to modulate the activity of primary sensory neurons (PSNs) in the DRG. However, not all patients receive adequate pain relief from DRGS (5–7), leaving them with few other treatment options. Unfortunately, complications, such as electrode lead migration, have further limited the success of DRGS (8, 9). Improving the clinical implementation of DRGS may help reduce the enormous burden of chronic pain.

One of the factors precluding our ability to improve DRGS is that we do not understand the physiological mechanisms of action behind DRGS-induced pain relief (10). Computational studies suggest that DRGS likely drives the activity of Aβ-neurons (11, 12), possibly driving feed-forward pain-gating circuitry in the spinal cord dorsal horn (13, 14). Work from other computational and preclinical studies suggests that DRGS may directly activate C-neurons (15–18), possibly augmenting the low-pass filtering of orthodromically propagating action potentials (APs) at the T-junction. However, it is well known that extracellular electrical stimulation, like that utilized by DRGS, preferentially activates myelinated axons over nonmyelinated axons and cell bodies (19–22). Computational studies of clinical DRGS corroborate this notion. Model Aβ-neuron activation thresholds (i.e., the current amplitude needed to generate an AP) have been reported on the order of several hundred μA (11, 12), whereas model C-neuron activation thresholds have been reported on the order of several mA (11, 12, 16). Clinically, therapeutic DRGS is typically applied with pulse amplitudes less than 1 mA (5, 23). This trend suggests that Aβ-neurons may be the predominant substrate of DRGS-induced pain relief.

However, a recent study using a rat model of DRGS demonstrated that when stimulating a DRG, the activation thresholds of C-neurons were only 1.5 times greater than the activation thresholds of Aβ-neurons (17). Though these data contrast with conventional neurostimulation intuition, they also suggest that there may be features unaccounted for in previous computational modeling studies that produce C-neurons with activation thresholds comparable to those of Aβ-neurons. Our previous work parametrized a single C-neuron model (i.e., determined one set of maximal ion channel conductances which reproduced AP characteristics measured in the experimental literature) (11, 12). However, transmembrane ion channels are under constant turnover, and neurons in the same population (e.g., C-neurons) which have similar electrophysiological characteristics may have different expression profiles of voltage-gated ion channels (24). It is possible that C-neurons with different expression levels of voltage-gated ion channels have significantly different activation thresholds in response to extracellular stimulation. Furthermore, studying the effects of DRGS on a population of biophysically distinct neurons may reveal differences across cells in how DRGS affects AP propagation through the T-junction (25).

To understand the possible effects of DRGS on T-junction filtering in C-neurons, it is critical to understand how biophysical parameters, such as ion channel expression, affect the orthodromic propagation of APs through the T-junction of C-neurons. A previous computational study of C-neurons showed that KCNQ channels, high-voltage activated L-type calcium channels (i.e., Ca_L_ channels), and small-conductance calcium-activated potassium channels (i.e., SK channels) can lower the maximum frequency at which APs propagate through the T-junction (i.e., the following frequency) (26). Specifically, inclusion of these channels in a C-neuron model was able to reduce the model's following frequency to less than 10 Hz, similar to following frequencies reported in experimental literature (15, 27, 28). However, those experimentally measured following frequencies were calculated by initiating APs in the axonal process which projects to the spinal cord (i.e., the spinal axon) (15, 27, 28), in accordance with previous studies in embryonic DRG neurons (29, 30).

The work of Lüscher and colleagues suggested that the following frequency of APs propagating through the T-junction in embryonic C-neurons is equivalent for both orthodromically propagating and antidromically propagating APs (29, 30). However, the diameter of the spinal axon of C-neurons is notably smaller (approximately half the size) than the diameter of the peripheral process (31–33). This crucial anatomical feature of C-neurons may not have been accounted for by the experiments on embryonic cells of Lüscher and colleagues, as axonal diameter likely changes through development (30). It is well understood that an AP is more likely to fail to propagate through a region transitioning from a smaller to larger diameter axon than a region transitioning from a larger to smaller diameter axon (34, 35). When an AP approaches a region of an axon with a decrease in diameter, the AP waveform at the branch increases in amplitude and occurs sooner compared to an axon that does not change diameter (34). This trend suggests that experimentally measured antidromic following frequencies in adult neurons (15, 27, 28) may not be representative of the following frequencies of orthodromically propagating APs. Therefore, subsequent studies of DRGS on C-neuron models that were parametrized to have orthodromic following frequencies that reproduced experimental antidromic following frequencies may be overestimating the effects of DRGS on T-junction filtering (16). To understand how DRGS may affect the orthodromic propagation of APs into the central nervous system, we must first understand how morphological and electrophysiological features of DRG neurons affect AP propagation through the T-junction.

The goal of this work was to investigate how DRGS affects the activity of a population of biophysically distinct C-neurons. The rationale for this work was that accounting for inherent biological variability in a population of model C-neurons may shed light on which biophysical parameters influence T-junction filtering. To simulate the neural response to DRGS, we coupled a finite element method (FEM) model of DRGS to a multi-compartment model of a C-neuron. We used a Markov Chain Monte Carlo (MCMC) method to generate one thousand unique combinations of maximal ion channel conductances with which to parameterize the multi-compartment C-neuron model. Our results suggest that across a population of biophysically distinct model C-neurons, the stimulus pulse amplitudes required to generate APs in C-neurons (i.e., ≥4 mA) are outside the ranges typically used clinically (i.e., ≤1 mA). However, applying DRGS at pulse amplitudes greater than model C-neuron activation threshold augmented T-junction filtering in some model C-neurons.

## Materials and methods

### FEM model of DRGS

**Figure 1:**
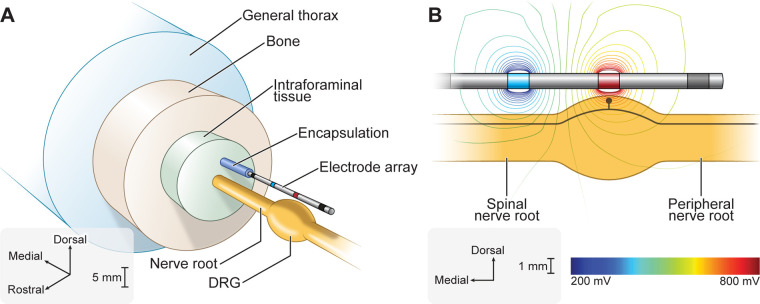
Finite element method (FEM) model of DRGS. We developed an FEM model of a human L5 DRG and surrounding anatomy. We implemented our previously published FEM model of DRGS (12). We included a model of a four-contact DRGS electrode array oriented above the DRG. (**A**) Exploded view of the DRGS FEM model. (**B**) We used the FEM model to calculate the extracellular potentials generated by DRGS. The trajectory of a model C-neuron is indicated by the black trace within the DRG.

We implemented our previously published FEM model of a human L5 DRG with an explicit representation of a clinical four-contact cylindrical DRGS electrode array (Figure 1A) (12). The geometry of the model was based on imaging and cadaver studies of human DRG and the surrounding anatomy (36–39). We set the conductivity of each tissue to the values used in our previous studies of DRGS (11, 12). We modeled all conductivities as isotropic, with the exception of the white matter in the nerve roots. We built the FEM model in the commercially available 3-matic module within the Mimics Innovation Suite (Materialise, Brussels, Belgium). We oriented the electrode array above the DRG such that the middle of the active contact (the second contact relative to the tip of the electrode array) was oriented above the midpoint of the DRG. We surrounded the electrode array with a 300 μm thick encapsulation layer to represent the foreign body response to implanted objects (40).

We imported the FEM model into COMSOL Multiphysics (COMSOL, Inc., Burlington, MA, USA). We simulated bipolar DRGS by applying a unit current stimulation boundary condition (i.e., 1 A) to the active electrode contact and grounded (i.e., 0 V) the return contact. We modeled the electrode lead shaft as a perfect insulator and modeled inactive electrode contacts as equipotential with zero net current across their surface. To calculate the potential distribution generated by DRGS (Figure 1B), we used the conjugate gradient method to solve Laplace's equation. To calculate model impedances, we divided the average voltage measured at the surface of the active contact by the applied stimulus current. The FEM model reproduced bipolar impedances similar to those reported clinically (i.e., 1,459 ± 715 Ω) (23).

### Multi-compartment C-neuron model

**Figure 2:**
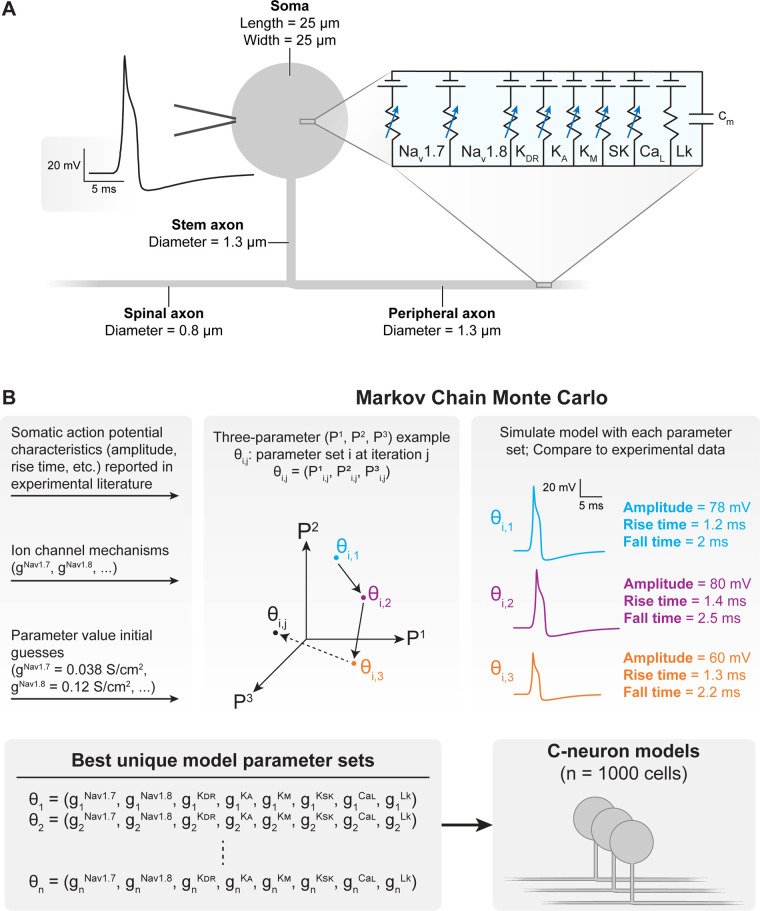
Construction and parametrization of a population of biophysically distinct multi-compartment model C-neurons. (**A**) We implemented a modified version of our previously published multi-compartment C-neuron model (11, 12). A representative action potential (AP) is shown on the left, and the included active ion channels are shown in the circuit schematic on the right. (**B**) We implemented a Markov Chain Monte Carlo (MCMC) method to parametrize a population of model C-neurons with different combinations of maximal ion channel conductances (41). A three-parameter (P1–P3) example is shown for visualization purposes. Our implementation of the MCMC method simulated thousands of possible parameter combinations (i.e., gNav1.7, gNav1.8, gKDR, gKA, gKM, gKSK, gCaL, gLk) in the model C-neuron and compares the resulting AP characteristics to those published in experimental literature. The result of the MCMC method was a population of the one thousand “best” parameter combinations which we could simulate in our model C-neuron during DRGS.

We modified a previously published model of a C-neuron (Figure 2A) (11). The model was validated on its ability to reproduce somatic AP characteristics measured experimentally (11, 27, 42, 43). The general morphology of the model was based on a previously published model of a C-neuron (26). We placed the model C-neuron within the FEM model such that its soma was located beneath the active DRGS electrode contact near the dorsomedial edge of the ganglion, as human DRG have the highest density of somata in the dorsomedial region of the ganglion (44).

We made several modifications to the morphology of the model. First, we set the diameters of the centrally projecting axon and peripherally projecting axon to 0.8 and 1.3 μm, respectively. These values are closer to the average values measured from cat DRG (31). Furthermore, larger diameter axons have lower activation thresholds than smaller diameter axons (19, 20, 22), improving the likelihood that we would see C-neuron activation in response to DRGS. We also decreased the stem axon diameter from 1.4 to 1.3 μm to better reproduce experimentally measured following frequencies. The following frequency is the maximum frequency at which APs can propagate through a branch point (e.g., the T-junction). Finally, we shortened the stem axon from 869 to 150 μm, to increase potential electrotonic effects of hyperpolarization at the soma on the T-junction (the proposed mechanism of augmented T-junction filtering) (16, 26). In some simulations, we varied the diameter of the spinal (i.e., centrally projecting) axon, to determine how the relative diameters of the spinal, peripheral, and stem axons affected AP propagation through the T-junction. Unless otherwise stated, we set the diameter of the spinal axon to 0.8 μm.

We also made changes to the active ion channels included in the model. We included several ion channel mechanisms represented in a previous C-neuron model that may be critical in producing T-junction filtering (16, 26). Specifically, we included representations of the outward potassium M-current, an L-type voltage-gated calcium channel, and the small conductance calcium-activated potassium channel (SK) (26). Typically, these additional channels were only expressed in the soma. However, where noted below, we also included these ion channels in all stem axon compartments and the first 50 compartments of the peripheral and central axons closest to the T-junction as described previously (26). We also removed the representation of Nav1.9 from our model to reduce computational complexity and dimensionality during model population generation. We set the decay time constant of intracellular calcium concentration to 5 s to reflect values measured in DRG neurons (45). None of these modifications produced significant effects on the AP characteristics (e.g., amplitude, duration) used to validate the original model.

### Parametrizing the C-neuron population

We implemented Goodman and Weare's Affine-Invariant Markov Chain Monte Carlo (MCMC) method using the emcee Python package (https://emcee.readthedocs.io) to generate a population of C-neuron models each with a unique set of maximal ion channel conductances (Figure 2B) (46, 47). Previous studies have used this approach to parametrize cable models of neurons, either by estimating the values of several ionic current parameters (48), or generating *de novo* combinations of maximal ion channel conductances to parametrize a population of models (41). Typically, MCMC methods use Bayes’ theorem to estimate posterior probabilities of a set of parameter values describing a given system based on prior assumptions about the distribution of each parameter's values and experimental data. We assumed uniform distributions for each parameter as priors, and we constrained each distribution relative to physiologic ranges (e.g., conductances must be greater than or equal to zero). In this work, we implemented the MCMC method to simulate and validate thousands of possible parameter sets (i.e., combinations of maximal ion channel conductances) (41). Each parameter set was evaluated on its ability to produce somatic AP characteristics (e.g., amplitude, duration) described by previous preclinical experiments with C-neurons (Table 1) (27, 28, 42, 43). We calculated the normalized distance between each parameter set's AP characteristic values and the mean values reported by previous experiments. We averaged the normalized distance across all metrics to calculate a “score” for each parameter set. A lower score indicated that a parameter set produced model AP characteristics that were closer to the mean of the experimentally measured characteristics’ values. We selected 1,000 models with the lowest scores for analysis in this study.

**Table 1:** Model and experimental ranges for somatic action potential validation metrics.

	Model population range (min, max)	Model population (mean ± SD)	Experimentally measured values	References
Amplitude (mV)	(65.5, 87.1)	72.8 ± 3.4	Median: 75 25th percentile: 64 75th percentile: 85	(27)
Duration (base) (ms)	(3.16, 4.71)	3.63 ± 0.27	Mean: 4.97 SD: 2.2	(42)
Rise time (ms)	(1.18, 2.70)	1.88 ± 0.30	Median: 2.0 Minimum: 0.8 Maximum: 5	(27)
Fall time (ms)	(1.58, 2.54)	1.75 ± 0.10	Median: 3.5 Minimum: 1.5 Maximum: 10.5	(27)
AHP amplitude (mV)	(6.1, 10.6)	9.6 ± 0.7	Mean: 8.2 SD: 5.1	(42)
AHP 80% recovery (ms)	(13.7, 26.9)	18.1 ± 1.7	Mean: 14.4 SD: 9.2	(43)

AHP, afterhyperpolarization; SD, standard deviation.

To ensure the MCMC method fully explored each parameter space, we ran the MCMC algorithm four times, with each successive run injecting progressively increasing variance of gaussian noise in the initial guess for each parameter value. Each run utilized 400 individual Markov chains (i.e., “walkers”) which were allowed to iterate their parameter values 25 times per run (41).

### Following frequency

We were interested in how biophysical parameters (e.g., axon diameter, ion channel conductance) affected AP propagation through the T-junction of model C-neurons. To mimic previous experimental methods, we defined a model's following frequency as the maximum frequency at which a model C-neuron can propagate a train of 20 APs through the T-junction (15). We calculated following frequencies through the T-junction in both the orthodromic (i.e., peripheral axon to spinal axon) and antidromic (i.e., spinal axon to peripheral axon) directions. We calculated following frequencies by initiating 20 APs in a given axon and recording the maximum rate at which all 20 APs successfully propagated into the corresponding axon on the other side of the T-junction. For example, when calculating the orthodromic following frequency, we initiated 20 APs in the peripheral axon, and recorded the maximum frequency at which all 20 APs propagated into the spinal axon. We calculated following frequencies using a binary search algorithm with a precision of 1 Hz.

### Simulating the neural response to DRGS

**Figure 3:**
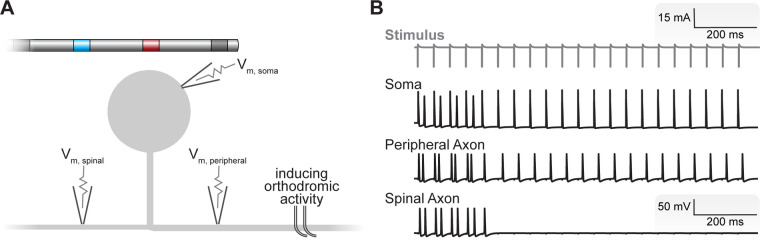
Simulating the neural response to DRGS. (**A**) We applied DRGS to each C-neuron model and recorded the resulting time-varying transmembrane voltages at the soma, peripheral axon, and spinal axon. In many cases, we were interested in how DRGS affected ongoing orthodromic activity (i.e., APs propagating from the peripheral axon, through the T-junction, to the spinal axon). A schematic of the four-contact DRGS electrode array is shown with the active contact (red) oriented above the soma of the model C-neuron (not to scale). (**B**) Time-varying transmembrane voltage traces in response to DRGS in a representative model C-neuron. The gray trace at the top represents the applied DRGS stimulus pulse. The black traces represent the time-varying membrane voltages of the soma, peripheral axon, and spinal axon. We initiated orthodromically propagating APs in the peripheral axon. Double APs are produced by closely timed APs generated by the DRGS pulse with those propagating orthodromically. In this representative neuron, suprathreshold DRGS induced T-junction filtering, blocking the propagation of APs into the spinal axon after a brief wash-in period putatively *via* the electrotonic spread of the slight hyperpolarization produced at the soma by DRGS.

We interpolated the extracellular potentials calculated by the FEM model onto the center of each neural compartment in our C-neuron model using the NEURON simulation environment within the Python programming language (49, 50). We simulated the time-varying extracellular potentials resulting from the output of the implantable DRGS pulse generator as described previously (11, 12, 51). We calculated each compartment's time-varying membrane voltage in response to DRGS (Figures 3A,B) using a backward Euler implicit integration method with a time step of 5 μs. We calculated activation thresholds, the minimum pulse amplitude required to elicit one or more action potentials in a C-neuron model, using a binary search algorithm with a precision of 0.1 μA. We calculated the activation threshold in response to one, three, or five DRGS pulses. In each case, we calculated the amplitude required to induce one-to-one activation, i.e., each applied pulse elicits one AP from the model C-neuron. We simulated DRGS pulses with a 300 μs pulse width because the majority of patients receiving DRGS to manage their chronic pain utilize a pulse width at or near 300 μs (23). Unless otherwise stated, we simulated DRGS pulses applied at a pulse frequency of 20 Hz because the majority of patients receiving DRGS to manage their chronic pain utilize pulse frequencies at or near 20 Hz (23).

In some simulations, we were interested in how tonic DRGS affected the orthodromic propagation of APs through the T-junction (Figure 3B). To study the effects of DRGS on AP propagation through the T-junction, we initiated 20 APs at 20 Hz in the peripheral axon of each C-neuron model and counted how many APs successfully propagated along the spinal axon. We then compared the number of APs propagating into the spinal axon of each C-neuron model across several test conditions (e.g., during subthreshold versus suprathreshold DRGS). We simulated the effects of one second of DRGS on a 20 Hz train of 20 orthodromically propagating APs.

In some simulations, we simulated the effects of γ-aminobutyric acid (GABA) on AP propagation in C-neurons. Recent work in rodents demonstrated that neurons which express 200 kDa neurofilament (i.e., large diameter myelinated afferents, such as Aβ-neurons) are capable of synthesizing and packaging GABA into synaptic vesicles (52). In simulations modeling the effects of GABA during DRGS, we assumed that each applied DRGS pulse activated an Aβ-neuron causing the release of GABA. We modeled somatic GABA conductances in each model C-neuron, which activated 1 ms after each DRGS pulse. We modeled the temporal dynamics (i.e., rise and decay time constants) of GABAergic currents as described previously (53). In DRG neurons, intracellular chloride concentrations are comparatively higher than in other regions of the nervous system. In primary afferents, the reversal potential of chloride is typicaly greater than a cell's resting membrane potential, causing GABA to produce a depolarizing effect (52, 54, 55). We set the reversal potential of GABA to −30 mV (56). We tuned the conductance of the GABA channel such that it produced inward currents of approximately 1 nA (52).

### Statistical analysis

We performed statistical analysis using the “stats” module within the SciPy Application Programming Interface (API) (https://scipy.org) within the Python programming language. When comparing two groups, we used the nonparametric Mann-Whitney *U* rank test. When examining correlations between biophysical parameters and resulting metrics (e.g., activation thresholds), we performed linear least-squares regression using the “linregress” method within the SciPy API. We established statistical significance with a threshold of *p* < 0.001.

## Results

### Model C-neuron population

Using the MCMC method, we developed a population of 1,000 biophysically distinct model C-neurons (i.e., each model C-neuron was parametrized with different combinations of maximal ion channel conductances). We first validated that our population of model C-neurons reproduced electrophysiological characteristics of C-neuron populations reported in the experimental literature. We compared the somatic AP characteristics (e.g., AP amplitude, duration) of our model C-neuron population with those measured experimentally. Table 1 reports the ranges, means, and standard deviations of our model C-neuron population's somatic AP characteristics, and compares them to the metrics reported in the experimental literature. In general, our model C-neuron population did an excellent job reproducing the somatic AP characteristics reported in the experimental literature.

### Effect of spinal axon diameter on AP propagation through the T-junction

The diameter of spinal axons of DRG neurons are approximately half the diameter of their peripheral axons (31, 33, 57). APs propagate through branch points with step decreases in diameter more readily than through branch points with step increases in diameter (34, 35). Therefore, DRG neuron morphology may facilitate AP propagation into the spinal axon from the peripheral or stem axons while limiting propagation from the spinal axon into the peripheral axon. Furthermore, it is hypothesized that DRGS may directly stimulate cell bodies (16, 58), suggesting DRGS may generate APs propagating in the stem axon towards the T-junction. We examined how the diameter of the spinal axon affected following frequencies through the T-junction in the orthodromic (i.e., peripheral axon to spinal axon), antidromic (i.e., spinal axon to peripheral axon), and somatic (i.e., APs initiated in the soma and propagating into the spinal axon) directions.

**Figure 4:**
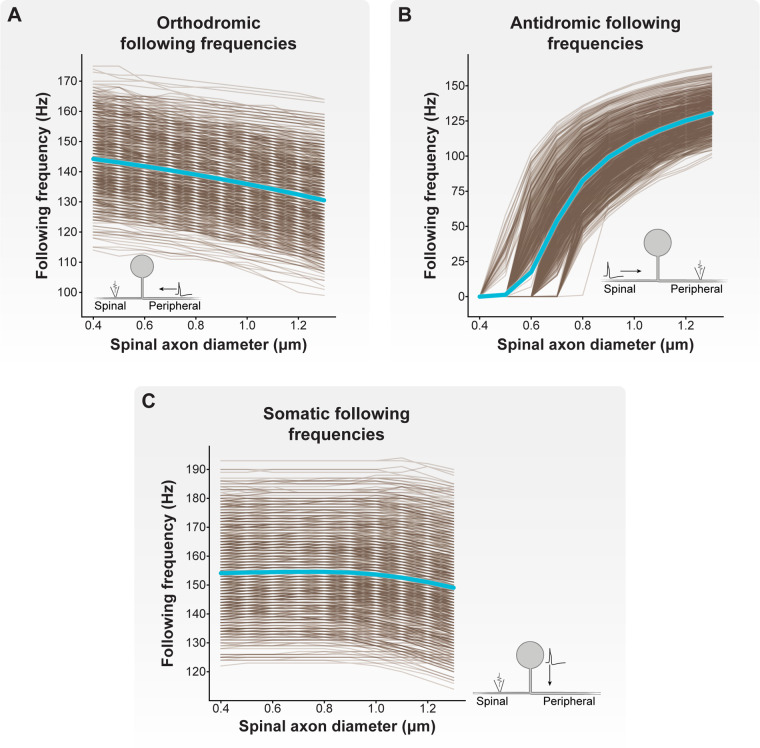
Effect of spinal axon diameter on following frequency. We varied the spinal axon diameter of each model C-neuron from 0.4 to 1.3 μm (note: the diameters of the peripheral and stem axons were 1.3 µm). Each brown line corresponds to an individual model C-neuron, and each blue line corresponds to the model population average. Inset cartoon schematics indicate for each plot where APs were initiated, the direction of propagation, and where we recorded APs in each model C-neuron. (**A**) Increasing the spinal axon diameter reduced orthodromic (i.e., peripheral axon to spinal axon) following frequencies. (**B**) Increasing spinal axon diameter increased antidromic (i.e., spinal axon to peripheral axon) following frequencies. (**C**) Increasing spinal axon diameter decreased somatic (i.e., from soma and stem axon to spinal axon) following frequencies.

Figure 4 demonstrates how increasing the diameter of the spinal axon from 0.4 to 1.3 μm affects orthodromic (Figure 4A), antidromic (Figure 4B), and somatic (Figure 4C) following frequencies across the C-neuron model population (note: the diameters of the peripheral and stem axons were 1.3 µm). When the spinal axon diameter was set to 0.4 µm, the orthodromic following frequency ranged from 114 to 175 Hz, with an average frequency of 144 Hz. The antidromic following frequency across all models was 0 Hz. And the somatic following frequency ranged from 122 to 193 Hz, with an average frequency of 154 Hz.

In general, increasing spinal axon diameter decreased orthodromic and somatic following frequencies, but increased antidromic following frequencies. When the spinal axon diameter was set to 1.2 µm (i.e., nearly equal to the 1.3 µm diameters of the peripheral and stem axons), the orthodromic following frequency ranged from 100 to 166 Hz, with an average frequency of 132 Hz. The antidromic following frequency ranged from 90 to 161 Hz, with an average of 125 Hz. And the somatic following frequency ranged from 117 to 192 Hz, with an average frequency of 151 Hz. Importantly, when the spinal axon diameter was set to 1.3 μm (i.e., equal to the peripheral and stem axon diameters), the minimum, maximum, and average antidromic and orthodromic following frequencies were equal. These results suggest that the relative diameters of the spinal, peripheral, and stem axons differentially affect the propagation of APs through the T-junction depending on where the APs initiate. Furthermore, these results suggest that spinal axons with diameters less than those of the peripheral or stem axons facilitate reliable orthodromic propagation of APs into the spinal axon.

### Effect of axonal conductance on AP propagation through the T-junction

Thus far, our results demonstrate that the diameter of the spinal axon affects the following frequency through the T-junction in all directions. However, our calculated following frequencies were generally larger (>100 Hz) than previously reported experimental measurements (consistently <100 Hz) (15, 27–29, 59). Previous modeling work suggested that the presence of slow active conductances (e.g., SK) in the axons may reduce following frequencies (26). Therefore, we next calculated orthodromic and antidromic following frequencies with the presence of L-type calcium channels and SK conductances in the stem axon and the 50 compartments proximal to the T-junction in the spinal and peripheral axons. To the best of our knowledge, there are no experimental reports of somatic following frequencies. Therefore, we excluded somatic following frequencies from subsequent analyses.

**Figure 5:**
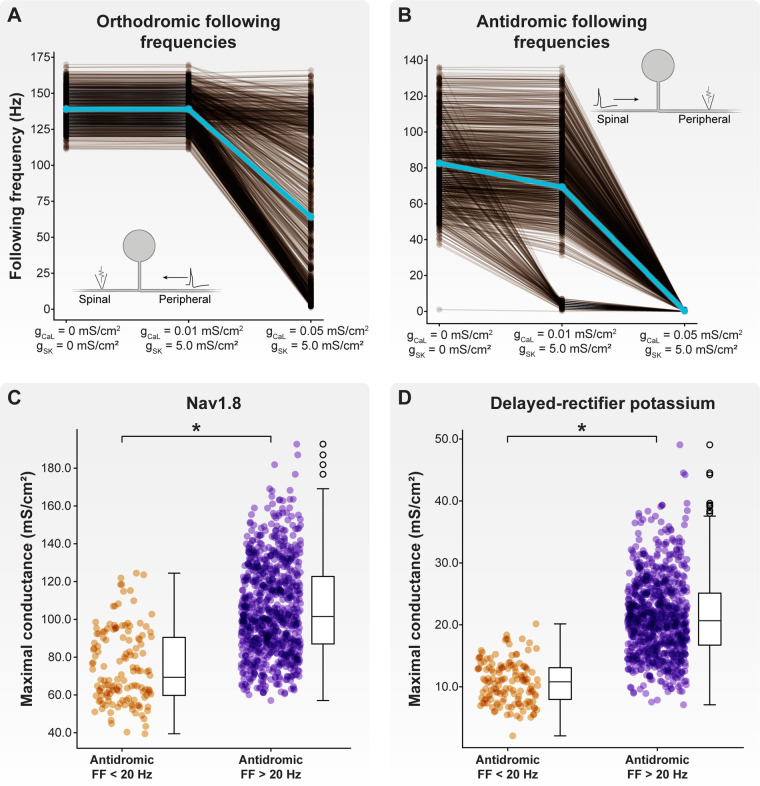
Effect of axonal SK and Ca_L_ conductances on following frequency. We fixed the maximal SK conductance at 5 mS/cm^2^ and increased Ca_L_ conductance from 0.01 to 0.05 mS/cm^2^. (**A**) Increasing maximal Ca_L_ conductance to 0.05 mS/cm^2^ produced a population of model C-neurons which displayed a wide range of orthodromic following frequencies. (**B**) Setting maximal Ca_L_ conductance to 0.01 mS/cm^2^ produced a population of model C-neurons which displayed a wide range of antidromic following frequencies. At this level of maximal Ca_L_ conductance, there appear to be two clusters of model C-neurons: those with antidromic following frequencies greater than 20 Hz, and those with antidromic following frequencies less than 20 Hz. Increasing maximal Ca_L_ conductance to 0.05 mS/cm^2^ dramatically reduced antidromic following frequencies. Model C-neurons with antidromic following frequencies greater than 20 Hz had significantly higher maximal (**C**) Nav1.8 and (**D**) delayed-rectifier potassium conductances than model C-neurons with antidromic following frequencies less than 20 Hz.

We first examined how the inclusion of axonal Ca_L_ and SK conductances affected orthodromic following frequencies (Figure 5A). Without including axonal Ca_L_ and SK conductances, the orthodromic following frequency of our model C-neuron population (with a 0.8 μm spinal axon) ranged from 111 to 170 Hz, with an average orthodromic following frequency of 139 Hz. When including a maximum Ca_L_ axonal conductance of 0.01 mS/cm^2^ and maximum SK axonal conductance of 5 mS/cm^2^, the orthodromic following frequency ranged from 111 to 170 Hz, with an average frequency of 139 Hz. When including a maximum Ca_L_ axonal conductance of 0.05 mS/cm^2^ and maximum SK axonal conductance of 5 mS/cm^2^, the orthodromic following frequency ranged from 1 to 166 Hz, with an average frequency of 64 Hz.

Next, we examined how the inclusion of axonal Ca_L_ and SK conductances affected antidromic following frequencies (Figure 5B). Without including axonal Ca_L_ and SK conductances, the antidromic following frequency ranged from 1 to 136 Hz, with an average frequency of 83 Hz. When including a maximum Ca_L_ axonal conductance of 0.01 mS/cm^2^ and maximum SK axonal conductance of 5 mS/cm^2^, the antidromic following frequency ranged from 0 to 136 Hz, with an average frequency of 69 Hz. When including a maximum Ca_L_ axonal conductance of 0.05 mS/cm^2^ and maximum SK axonal conductance of 5 mS/cm^2^, the antidromic following frequency ranged from 0 to 1 Hz, with an average frequency below 1 Hz. Taken together, these data suggest that axonal Ca_L_ and SK conductances can reduce orthodromic and antidromic following frequencies to ranges observed experimentally.

When including axonal Ca_L_ and SK conductances of 0.01 and 5 mS/cm^2^, respectively, there appeared to be two distinct clusters of model C-neurons: those with antidromic following frequencies greater than 20 Hz, and those with antidromic following frequencies less than 20 Hz (Figure 5B). We compared the biophysical parameters used to parametrize the models in these two groups. Interestingly, model C-neurons with antidromic following frequencies greater than 20 Hz had significantly higher maximal conductances of Nav1.8 (Figure 5C; MannWhitney *U* rank test, *p* < 0.001) and delayed-rectifier potassium channels (Figure 5D; MannWhitney *U* rank test, *p* < 0.001) relative to model C-neurons with antidromic following frequencies less than 20 Hz. These data suggest that fast-acting sodium and potassium channels may be key determinants in setting a C-neuron's following frequency.

### Effect of physiologic variability on C-neuron activation thresholds

Next, we examined how activation thresholds varied across our population of biophysically distinct model C-neurons. In these simulations, we calculated activation thresholds in the absence of axonal Ca_L_ and SK conductances. We calculated activation thresholds in response to anodic-first (i.e., a positive-current active phase) and cathodic-first (i.e., a negative-current active phase) stimulus pulses. We were also interested in how the number of pulses used to define the activation threshold affected the calculated value of the threshold. Therefore, we calculated the minimum current necessary to induce one-to-one activation (i.e., each applied stimulus pulse elicits one or more APs) in response to one, three, or five DRGS stimulus pulses.

**Figure 6:**
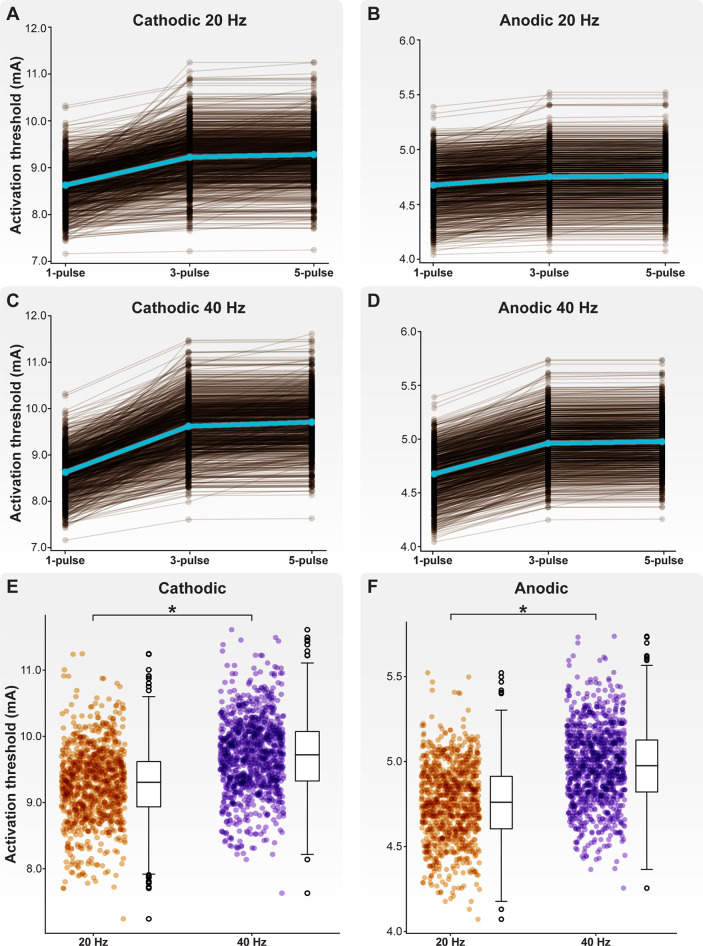
Activation thresholds of the model C-neuron population. Activation thresholds during (**A**) cathodic-first DRGS applied at 20 Hz, (**B**) anodic-first DRGS applied at 20 Hz, (**C**) cathodic-first DRGS applied at 40 Hz, and (**D**) anodic-first DRGS applied at 40 Hz. We calculated the threshold DRGS amplitude to produce one-to-one activation (i.e., every applied pulse generates at least one AP in a given cell) in response to one, three, and five DRGS pulses. Each brown line corresponds to an individual model C-neuron, and each blue line corresponds to the model population average. Five-pulse activation thresholds in response to 40 Hz DRGS were significantly higher than activation thresholds in response to 20 Hz DRGS for both (**E**) cathodic-first and (**F**) anodic-first DRGS.

Across all model C-neurons and threshold calculations, the cathodic-first activation threshold ranged from 7.16 to 11.61 mA. In response to cathodic-first stimulation, APs initiated a stem axon compartment proximal to the soma. Across all model C-neurons and all threshold calculations, the anodic-first activation threshold ranged from a minimum of 4.04 mA to a maximum of 5.74 mA. In response to anodic-first stimulation, APs initiated in the T-junction or in an axonal compartment proximal to the T-junction. In response to 20 Hz DRGS, the mean cathodic-first one-, three-, and five-pulse thresholds were 8.63 ± 0.45, 9.23 ± 0.56, and 9.28 ± 0.55 mA, respectively (Figure 6A). The mean anodic-first one-, three-, and five-pulse thresholds were 4.68 ± 0.22, 4.75 ± 0.22, and 4.76 ± 0.22 mA, respectively (Figure 6C). In response to 40 Hz DRGS, the mean cathodic-first one-, three-, and five-pulse thresholds were 8.63 ± 0.45, 9.62 ± 0.59, and 9.71 ± 0.57 mA, respectively (Figure 6B). The mean anodic-first one-, three-, and five-pulse thresholds were 4.68 ± 0.22, 4.96 ± 0.23, and 4.98 ± 0.23 mA, respectively (Figure 6D). The five-pulse activation thresholds for both cathodic-first (Figure 6E) and anodic-first (Figure 6F) DRGS applied with a pulse frequency of 40 Hz were significantly higher than the five-pulse activation thresholds for anodic-first and cathodic-first DRGS applied with a pulse frequency of 20 Hz (MannWhitney *U* rank test, *p* < 0.001). We hypothesized that increased activation thresholds in response to increased pulse frequency was due to the activation of slow outward conductances (e.g., somatic SK) in response to the first pulse, which hyperpolarizes the neuron requiring greater pulse amplitude to induce subsequent activation. Shorter durations between successive pulses likely do not give the membrane sufficient time to return to its resting potential before another pulse is applied. In general, these results suggest that differences in ion channel expression profiles across neurons may considerably affect their activation thresholds in response to extracellular electrical stimulation. A detailed understanding of the voltage-gated ion channels expressed by on- and off-target neural subpopulations in a given tissue, and how these expression profiles may vary across disease etiologies, may be helpful in the design and clinical implementation of a neurostimulation therapy. Furthermore, these results indicate that C-neuron activation thresholds in response to DRGS are typically outside the ranges of stimulation amplitudes used clinically (i.e., typically ≤1 mA) (23).

**Figure 7:**
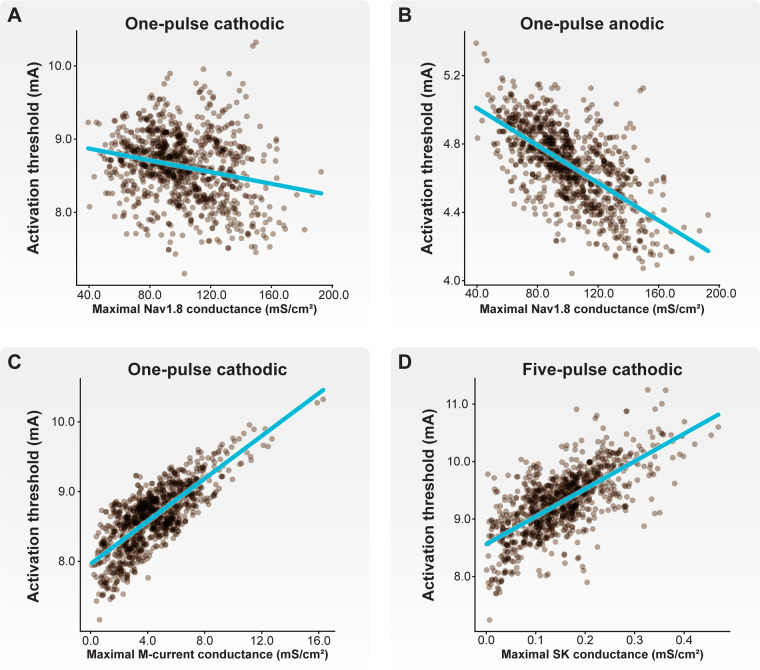
Correlations between biophysical parameters and activation thresholds. Each brown point corresponds to an individual model C-neuron. Each blue line corresponds to the linear least-squares regression. Both (**A**) cathodic-first and (**B**) anodic-first activation thresholds were negatively correlated with maximal Nav1.8 conductance. (**C**) Cathodic-first thresholds were correlated with maximal M-current conductance. (**D**) Five-pulse cathodic-first thresholds were correlated with maximal SK conductance.

Next, we examined how the maximal ionic conductances used to parametrize our C-neuron models correlated with activation thresholds (Figure 7). In general, increased Nav1.8 conductance significantly correlated with lower activation thresholds in response to cathodic- (Figure 7A; correlations for one-pulse thresholds: *p* < 0.001; *r* = −0.233) and anodic-first (Figure 7B; correlations for one-pulse thresholds: *p* < 0.001; *r* = −0.647) DRGS. Greater net conductance of fast-activating sodium channels (e.g., Nav1.8) likely reduces activation thresholds by producing greater inward currents in response to the active phase of the stimulus pulse. Increased M-current conductance significantly correlated with higher activation thresholds in response to cathodic-first DRGS (Figure 7C; correlations for one-pulse thresholds: *p* < 0.001; *r* = 0.771). The M-current is a key regulator of neural excitability, and conducts potassium out of the cell as a neuron approaches its AP threshold (60). Increased conductance of the channels underlying the M-current likely produces greater outward compensatory currents during the upstroke of a potential AP, raising the extracellular activation threshold as a consequence. Increased somatic SK conductance significantly correlated with cathodic-first five-pulse thresholds (Figure 7D; *p* < 0.001; *r* = 0.698). The slow outward current produced by SK channels likely does not affect a neuron's response to a single extracellular electrical impulse. Instead, the long-term hyperpolarization produced by SK channels likely requires greater stimulus pulse amplitudes for subsequent pulses to continually evoke APs. These results imply that the spatiotemporally varying expression levels of different ion channels may be crucial in setting a neuron's response to tonic extracellular electrical stimulation.

### Effect of axonal conductance on AP propagation during tonic DRGS

It is hypothesized that DRGS may provide pain relief by augmenting the filtering of APs at the T-junction of C-neurons (16, 17). Therefore, we next examined how tonic DRGS affected the propagation of APs through the T-junction in each model C-neuron. We initiated 20 APs at 20 Hz (26, 61) in the peripheral axon of each model C-neuron and applied one second of DRGS and observed how many APs successfully propagated through the T-junction (Figure 3B). We compared the number of successfully propagated APs across several conditions, such as inclusion of axonal Ca_L_ and SK conductances, with and without DRGS, and DRGS applied at subthreshold and suprathreshold stimulation amplitudes. When examining the effect of Ca_L_ and SK axonal conductances on AP propagation during DRGS, we compared maximal conductance values which produced a wide range of antidromic following frequencies (g_CaL_ = 0.01 mS/cm^2^; g_SK_ = 5 mS/cm^2^; Figure 5B) and orthodromic following frequencies (g_CaL_ = 0.05 mS/cm^2^; g_SK_ = 5 mS/cm^2^; Figure 5A).

**Figure 8:**
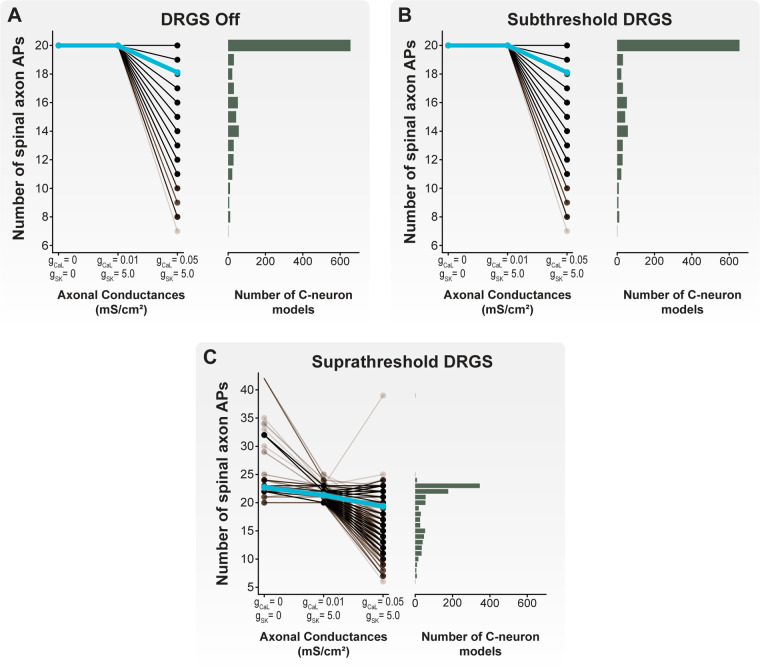
Effect of axonal Ca_L_ and SK conductances on AP propagation through the T-junction during tonic DRGS. Each brown line corresponds to an individual model C-neuron, and each blue line corresponds to the model population average. Horizontal histograms share the *y*-axis of each corresponding line plot and indicate the number of model C-neurons with a given number of APs propagating into its spinal axon. When (**A**) DRGS was turned off or (**B**) applied at 1 mA, including axonal SK and Ca_L_ conductances filtered some of the 20 APs propagating orthodromically through the T-junction. (**C**) When applying suprathreshold DRGS, some model C-neurons propagated more than the 20 peripherally generated APs into the spinal axon, while other model C-neurons filtered many of the APs generated in the periphery or by the DRGS pulse.

We first examined how the inclusion of Ca_L_ and SK conductances in the stem axon and in the spinal and peripheral axon compartments proximal to the T-junction affected orthodromic AP propagation through the T-junction during DRGS (Figure 8). We first simulated AP propagation through the T-junction when DRGS was turned off (i.e., the stimulus pulse amplitude was set to 0 mA) (Figure 8A). All 20 APs propagated into the spinal axon when there were no axonal Ca_L_ and SK conductances present. Similarly, when including axonal Ca_L_ and SK conductances which produced a wide range of antidromic following frequencies, all 20 orthodromic APs faithfully propagated into the spinal axon. When including axonal Ca_L_ and SK conductances which produced a wide range of orthodromic following frequencies, the number of APs which propagated through the T-junction in a given cell model ranged from 7 to 20 APs, with a population average of 18.1 APs. These data suggest that without applying DRGS, axonal Ca_L_ and SK conductances that produce a population of C-neurons with a wide range of orthodromic following frequencies are sufficient to produce T-junction filtering.

Next, we applied DRGS at a subthreshold amplitude of 1 mA (Figure 8B). It is important to note that we are using “subthreshold” to describe a stimulus pulse amplitude (i.e., 1 mA) which is insufficient to induce APs in all model C-neurons. However, DRGS applied at 1 mA is likely sufficient to generate APs in other neuron types, such as Aβ-neurons (11, 12), and produce paresthesias in patients (23, 62, 63). During subthreshold DRGS, all 20 APs propagated into the spinal axon when there were no axonal Ca_L_ and SK conductances present and when including axonal Ca_L_ and SK conductances that produced a wide range of antidromic following frequencies. When including axonal Ca_L_ and SK conductances which produced a wide range of orthodromic following frequencies, the number of APs which propagated through the T-junction in a given cell model ranged from 7 to 20 APs, with a population average of 18.1 APs. Therefore, these data suggest that applying DRGS at 1 mA (i.e., subthreshold DRGS relative to C-neuron thresholds) does not affect AP propagation through the T-junction compared to applying 0 mA DRGS under the same axonal conductance conditions.

We then applied DRGS at amplitudes necessary to generate one-to-one activation of each model C-neuron (i.e., suprathreshold DRGS) (Figure 8C). When excluding axonal Ca_L_ and SK conductances, the number of APs propagating into the spinal axon during suprathreshold DRGS ranged from 20 to 42 APs, with a population average of 22.7 APs. When including axonal Ca_L_ and SK conductances which produced a wide range of antidromic following frequencies in the model C-neuron population, the number of APs propagating into the spinal axon ranged from 20 to 25 APs, with a population average of 21.3 APs. When including axonal Ca_L_ and SK conductances which produced a wide range of orthodromic following frequencies in the model C-neuron population, the number of APs propagating into the spinal axon ranged from 6 to 39 APs, with a population average of 19.4 APs. In general, these data suggest that suprathreshold DRGS that induces activity in C-neurons, likely increases the number of APs propagating into the spinal axon. However, axonal Ca_L_ and SK conductances may be sufficient to reduce the number of APs propagating into the spinal axon during suprathreshold DRGS, similar to the effect of axonal Ca_L_ and SK conductances during subthreshold DRGS and when DRGS was not applied.

### Effect of stimulus pulse amplitude on AP propagation during tonic DRGS

**Figure 9:**
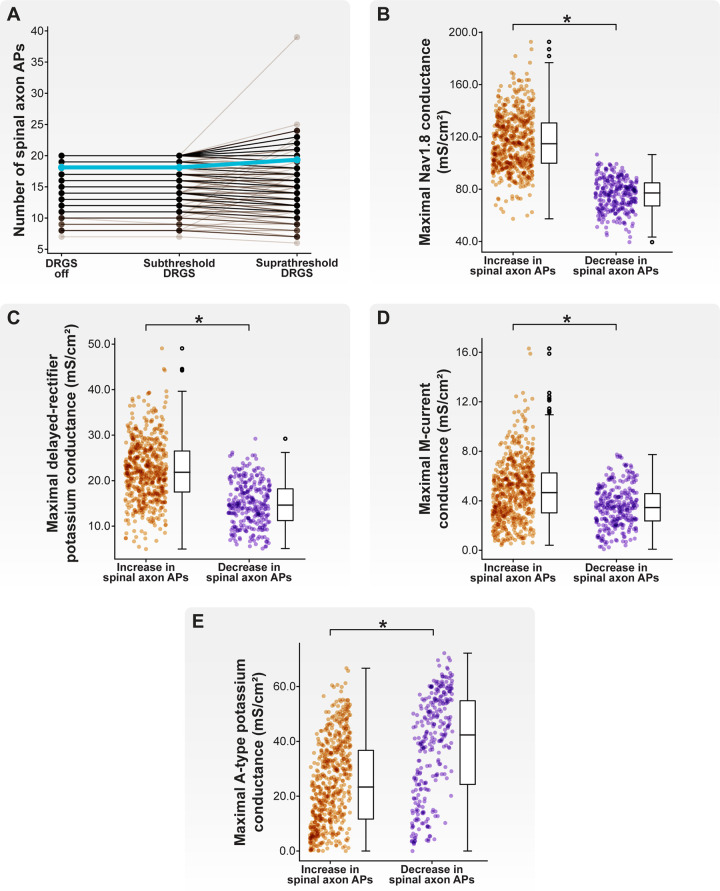
Effect of stimulus pulse amplitude on AP propagation through the T-junction during 20 Hz cathodic-first DRGS. (**A**) Each brown line corresponds to an individual model C-neuron, and each blue line corresponds to the model population average. Increasing the DRGS pulse amplitude from subthreshold to suprathreshold amplitudes caused some model C-neurons to increase the number of APs propagating through their T-junction, while others decreased the number of APs. (**B–E**) Comparing biophysical properties of model C-neurons which increased or decreased the number of APs propagating through their T-junctions in response to increasing DRGS pulse amplitude from a subthreshold to suprathreshold level. Model C-neurons which decreased the number of APs propagating through their T-junctions had lower maximal conductances of (**B**) Nav1.8, (**C**) delayed-rectifier potassium, and (**D**) M-current, but (**E**) higher maximal A-type potassium channel conductance.

Next, we examined the differences between cells that experienced an increase and cells that experienced a decrease in the number of APs propagating into the spinal axon when increasing DRGS amplitude (Figure 9). Our results suggest that when excluding axonal Ca_L_ and SK conductances or when using axonal Ca_L_ and SK conductances which reproduce antidromic following frequencies, DRGS is likely not affecting the number APs propagating into the spinal axon (Figure 8). Therefore, the model C-neurons in the remainder of our analysis will include axonal Ca_L_ and SK conductances which reproduce a wide range of orthodromic following frequencies (i.e., g_CaL_ = 0.05 mS/cm^2^; g_SK_ = 5 mS/cm^2^) (Figure 5A).

In general, there was no difference in the number of APs propagating into the spinal axon when increasing the DRGS pulse amplitude from 0 to 1 mA (Figure 9A). When applying DRGS with a pulse amplitude of 1 mA, model C-neurons propagated between 7 and 20 APs into their spinal axons. Increasing the DRGS pulse amplitude from 1 mA to a given model C-neuron's activation threshold caused some model C-neurons (598 of 1,000 models; 59.8%) to increase the number of APs propagating into their spinal axon, while other model C-neurons experienced a decrease (293 of 1,000 models; 29.3%) (Figure 9A). Some models (109 of 1,000 models; 10.9%) did not experience a change in the number of APs entering their spinal axons when increasing the DRGS pulse amplitude.

We next compared the biophysical properties between model C-neurons which increased and C-neurons which decreased the number of APs entering their spinal axons when increasing DRGS pulse amplitude from sub- to suprathreshold. Model C-neurons which experienced a decrease in the number of APs propagating into the spinal axon had lower maximal Nav1.8 (Figure 9B; MannWhitney *U* rank test, *p* < 0.001), delayed-rectifier potassium (Figure 9C; MannWhitney *U* rank test, *p* < 0.001), and M-current (Figure 9D; MannWhitney *U* rank test, *p* < 0.001) conductances than model C-neurons which experienced increases in the number of spinal axon APs. Lower Nav1.8 conductance likely prevents the membrane around the T-junction from depolarizing sufficiently to propagate an incoming AP through the branch point, while lower delayed-rectifier potassium conductance may prolong membrane repolarization and the refractory period around the T-junction. In contrast, model C-neurons which experienced a decrease in the number of APs propagating into the spinal axon had greater maximal KA conductances than model C-neurons which experienced an increase in the number of spinal axon APs (Figure 9E; MannWhitney *U* rank test, *p* < 0.001). The fast kinetics of A-type channels suggests that increasing their net conductance likely makes it more difficult for a single AP to propagate through the T-junction and also reduces the maximum rate at which the T-junction membrane is able to receive and propagate APs (64, 65). Taken together, these data suggest that increasing DRGS pulse amplitude from sub- to suprathreshold levels may increase the activity of some C-neurons while decreasing the activity of others. This notion underscores the importance of understanding which neural subpopulations generate APs in direct response to a stimulus pulse that mimics the waveforms utilized in clinical implementation of DRGS. Furthermore, differences in ion channel expression, particularly ion channels with fast kinetics or those active near a cell's AP threshold, may influence how C-neurons change their activity in response to sub- and suprathreshold DRGS.

### Effect of GABA on AP propagation through the T-junction

Recent experimental work demonstrated functional GABAergic signaling in rodent DRG (52). Interestingly, depolarizing GABAergic DRG neurons reduced nocifensive behavior, possibly by blocking spike propagation through the T-junction (52). Next, we examined how a somatic GABA conductance may affect the propagation of APs through the T-junction in each model C-neuron during tonic DRGS.

**Figure 10:**
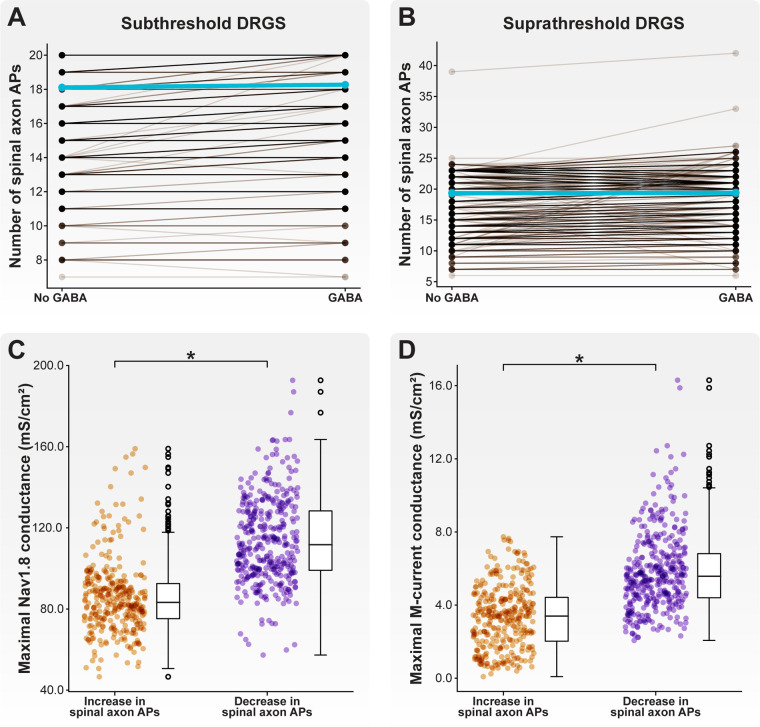
Effect of GABA on AP propagation through the T-junction during tonic DRGS. Each brown line corresponds to an individual model C-neuron, and each blue line corresponds to the model population average. Including a somatic GABA conductance activated 1 ms after the DRGS pulse caused some model C-neurons to increase the number of APs propagating through the T-junction while other model C-neurons decreased the number of APs during (**A**) subthreshold and (**B**) suprathreshold DRGS. Model C-neurons which decreased the number of APs entering their spinal axons had significantly higher maximal (**C**) Nav1.8 and (**D**) M-current conductances.

We first examined how a somatic GABA conductance may affect spike propagation through the T-junction during subthreshold DRGS (i.e., the pulse amplitude was set to 1 mA). Including a somatic GABA conductance during subthreshold DRGS differentially affected model C-neurons (Figure 10A). Some model C-neurons (135 of 1,000 models; 13.5%) experienced an increase in the number of spikes propagating into their spinal axons during subthreshold DRGS, while others (3 of 1,000 models; 0.3%) experienced a decrease. Many model C-neurons (862 of 1,000 models; 86.2%) saw no change in the number of spikes propagating into their spinal axons during subthreshold DRGS with a somatic GABA conductance. Including a somatic GABA conductance did not significantly affect the model population average number of spikes entering the spinal axon (18.1 spikes without GABA, 18.2 spikes with GABA).

We next examined how a somatic GABA conductance may affect spike propagation during suprathreshold DRGS. Like subthreshold DRGS, including a somatic GABA conductance during suprathreshold DRGS differentially affected different model C-neurons (Figure 10B). Some model C-neurons (335 of 1,000 models; 33.5%) experienced an increase in the number of spikes propagating into their spinal axons during suprathreshold DRGS, while others (373 of 1,000 models; 37.3%) experienced a decrease. Some model C-neurons (292 of 1,000 models; 29.2%) saw no change in the number of spikes propagating into their spinal axons. Including a somatic GABA conductance did not significantly affect the model population average number of spikes entering the spinal axon (19.4 spikes without GABA, 19.4 spikes with GABA).

Next, we compared the biophysical properties of model C-neurons that experienced an increase to C-neurons that experienced a decrease in the number of spinal axon APs propagating through the T-junction as a result of including a somatic GABA conductance during suprathreshold DRGS. Model C-neurons that experienced a decrease in spinal axon APs had higher maximal Nav1.8 conductances (Figure 10C; *p* < 0.001) and M-current conductances (Figure 10D; *p* < 0.001) than model C-neurons which experienced an increase in spinal axon APs. These data suggest that somatic GABA conductances may increase the activity of some C-neurons in response to subthreshold DRGS, but augment T-junction filtering in some C-neurons in response to suprathreshold DRGS. Higher expression levels of fast sodium channels (e.g., Nav1.8) and larger M-currents may mediate augmented T-junction filtering in response to suprathreshold DRGS.

## Discussion

In this work, we employed a computational modeling approach to studying the effect of DRGS on C-neurons. We first developed a population of one thousand similarly behaving, but biophysically distinct, multi-compartment cable models of C-neurons. We used this population of model C-neurons to study how morphological and electrophysiological characteristics of C-neurons affect the propagation of APs from the peripheral to the spinal axon. We found that smaller diameter spinal axons relative to peripheral axons enabled the propagation of higher frequency trains of orthodromic APs through the T-junction (Figure 4). We also demonstrated that slow outward conductances (e.g., SK) present in axonal compartments proximal to the T-junction may be sufficient to produce following frequencies within the ranges observed experimentally (Figure 5).

We then examined how DRGS affected a population of biophysically distinct cells. We found that the maximal conductances used to parametrize a model C-neuron can significantly affect the DRGS amplitude necessary to induce APs in the cell. In general, our data suggest that DRGS applied at stimulus pulse amplitudes used clinically (i.e., ≤1 mA) likely does not directly modulate the activity of C-neurons. However, DRGS applied at pulse amplitudes sufficient to generate APs in model C-neurons produces a diverse range of effects on C-neurons. In some model C-neurons, suprathreshold DRGS reduced the number of APs entering the spinal axon, while increasing the number of APs entering the spinal axon in other model C-neurons. In summary, the data presented in this manuscript suggest that inherent biological variability (e.g., in ion channel expression) may significantly affect both how a neuron responds to extracellular electrical stimulation (e.g., activation threshold), and how stimulation affects the propagation of ongoing afferent activity (e.g., the number of APs propagating through a branch point).

### Action potential propagation through the T-junction of C-neurons

The propagation of APs through the T-junction of C-neurons is a critical step in coding painful input into the central nervous system. Previous experimental work suggested that the rate at which APs can propagate through the T-junction in either direction (i.e., antidromic and orthodromic following frequencies) are comparable (29, 30). Subsequent studies calculated following frequencies in the antidromic direction as a proxy for orthodromic following frequencies (15, 27, 28). However, the spinal axons of DRG neurons are smaller than peripheral axons (31, 33, 57), and decreases in axon diameter should facilitate propagation through a branch point while increases in axon diameter should reduce propagation through a branch point (34, 35, 66). Therefore, orthodromic following frequencies should be greater than those measured in the antidromic direction.

Our results corroborate this notion. When simulating AP propagation through the T-junction, spinal axons with diameters smaller than the peripheral axon diameter produce orthodromic following frequencies greater than the corresponding antidromic following frequency (Figures 4A,B). Increasing the spinal axon diameter while holding the stem and peripheral axon diameters constant reduced orthodromic following frequencies and increased antidromic following frequencies (Figures 4A,B). Much of the experimental work reporting following frequencies through the T-junction measured following frequencies in the antidromic direction (15, 27, 28), or in embryonic cells which may still be undergoing development which leads to the diameter mismatch at the T-junction (30). Therefore, true ranges of orthodromic following frequencies in C-neurons may be unknown. Future experimental work measuring orthodromic following frequencies in adult tissue are needed to understand if the predictions of the models in this work are accurate, or if there are peculiarities of DRG neurons which facilitate conduction through the T-junction in both directions at similar rates. For example, mathematical models studying AP propagation through branch points assume homogeneous ion conductances around the branch point (34, 35). Understanding the spatiotemporal expression profiles of different ion channels near the T-junction of C-neurons would be useful in both understanding the propagation of APs through this junction, and in parametrizing multi-compartment cable models for future studies of DRGS.

The work of Stoney reported orthodromic following frequencies in frogs of approximately 90 Hz on average. The orthodromic following frequencies produced by our model C-neuron population were all greater than 90 Hz (Figure 4A). Previous work suggested that inclusion of slow active conductances (e.g., SK) in the axonal compartments near the T-junction reduces following frequencies (26). Furthermore, immunohistochemical data suggest that SK may be present in the axons of DRG neurons (67). We examined how including SK and Ca_L_ (which provided the calcium influx necessary to activate SK) conductances in the axonal compartments proximal to the T-junction affected following frequencies (Figure 5). We fixed the maximal axonal SK conductance at 5 mS/cm^2^, similar to previous work (26). Including an axonal Ca_L_ conductance of 0.01 mS/cm^2^ reduced antidromic following frequencies, without significantly affecting orthodromic following frequencies (Figures 5A,B). Further increasing the axonal Ca_L_ conductance to 0.05 mS/cm^2^ (putatively increasing the activation of SK channels) reduced almost all antidromic following frequencies to zero, but moderately reduced orthodromic following frequencies producing a wide range of following frequencies encompassing the ranges reported experimentally (Figures 5A,B). These data add further evidence across a population of model C-neurons that slow axonal conductances may be sufficient to produce T-junction filtering in DRG neurons. These data also suggest that further experimental work is needed to understand the relationship between orthodromic and antidromic following frequencies in adult C-neurons.

When including axonal conductances which produce a model C-neuron population with a wide range of antidromic following frequencies, we found two distinct clusters of models: those with antidromic following frequencies greater than 20 Hz, and those with antidromic following frequencies less than 20 Hz (Figure 5B). Interestingly, we found that model C-neurons in the cluster of antidromic following frequencies greater than 20 Hz had greater Nav1.8 (Figure 5C) and delayed-rectifier potassium (Figure 5D) maximal conductances than models with antidromic following frequencies less than 20 Hz. It is possible that greater sodium conductance can produce sufficient depolarization to facilitate AP propagation through a junction even when the geometric ratio of the branches is unfavorable (26, 35). Concurrently, a greater density of delayed-rectifier potassium channels may allow the neural membrane to repolarize more quickly, shortening the refractory period and preparing the membrane to allow an incoming AP to propagate through the T-junction. Continued study of how the interplay between different ion channels affects AP propagation through branch points is needed to understand how neurostimulation therapies affect AP propagation in morphologically complex neurons.

### Augmenting T-junction filtering in C-neurons as a mechanism of action of DRGS

It is hypothesized that a primary mechanism of DRGS-induced pain relief is the augmentation of AP filtering at the T-junction of C-neurons (10, 15–17). This hypothesis suggests that either DRGS is directly activating C-neurons to modulate or disrupt their ongoing activity, or DRGS is indirectly modulating the activity of C-neurons as a consequence of some other mechanism of DRGS. In this work, we examined how DRGS may be directly affecting C-neurons by simulating the effects of DRGS on a population of biophysically distinct C-neuron models.

If DRGS is directly activating C-neurons to augment T-junction filtering, the minimum current amplitude needed to induce APs in a C-neuron (i.e., the activation threshold) must be within the range of pulse amplitudes used clinically (i.e., typically ≤1 mA) (23). Across our model C-neuron population, the lowest activation threshold was 4.0 mA and the maximum activation threshold was 11.6 mA. These amplitudes are far outside of the typical clinical amplitudes and several times larger than the activation thresholds of model Aβ-neurons found in previous computational studies of DRGS (i.e., on the order of hundreds of μA) (11, 12). Furthermore, we modeled a single C-neuron location in the DRG, with its soma oriented directly below the active electrode contact (Figure 1B). C-neurons farther from the active electrode contact would likely have considerably larger activation thresholds (11). Therefore, the data presented in this manuscript suggest that the direct activation of C-neurons by the DRGS pulse is unlikely in clinical scenarios.

In contrast, a recent study of DRGS in a rat model suggested that the activation thresholds of C-neurons are only 1.5 times greater than the activation thresholds of Aβ-neurons (17). It is possible that the small electrodes used to stimulate the DRG in preclinical experiments may produce higher current densities relative to the current densities generated with clinical electrodes. Higher current densities from preclinical electrodes may be sufficient to lower the activation thresholds of C-neurons (68), but it is unclear if such trends would be present using state-of-the-art clinical systems. It is also possible that there are one or more features of C-neurons not accounted for by our models which substantially reduce activation thresholds. Such features could include complex axonal trajectories present in the stem axons of C-neurons (57, 69, 70), intra- and inter-neuronal ephaptic coupling (71), and cross-excitation between DRG neurons possibly mediated by satellite glial cells (72, 73). Continued study of the influence of these features on the activation of nonmyelinated neurons would be of great interest both to understanding the mechanisms of DRGS and to the broader neuromodulation community.

Though our data indicate clinical DRGS is likely not directly activating C-neurons, our data also suggest it is possible for DRGS to augment filtering in some C-neurons (Figures 3B, 8–10). However, our data indicate the extent to which DRGS augments filtering in C-neurons may vary across C-neurons in the population. These findings are at odds with experimental data, which indicate that augmented T-junction filtering is robust across many measured C-neurons at amplitudes comparable to those which activate Aβ-neurons (17). However, there are key differences between the simulations presented in this work and the published preclinical experiments. First, as described above, the larger current densities generated by preclinical electrode arrays compared to those generated by clinical electrode arrays may significantly reduce the stimulus pulse amplitudes needed to modulate the activity of C-neurons (68). Second, our data suggest that the presence of slow outward ionic conductances (e.g., SK) near the T-junction can significantly limit the propagation of APs into the spinal axon of C-neurons (Figure 5). However, there is a dearth of published data describing the spatial distribution and magnitudes of different ionic currents in DRG axons, particularly near the T-junction. Future experimental studies are needed to determine which ion channels are present near the T-junction, and how DRGS may activate these currents to augment T-junction filtering. Finally, to reduce computational demand, we were limited to simulating the effects of DRGS on C-neurons for approximately one second. However, the work of Chao and colleagues demonstrated robust activity dependent filtering, predominantly in C-neurons, after a wash-in period between 5 and 30 s (17). Therefore, the voltage-gated channels with kinetics which operate on the order of milliseconds like those used in this study may be capable of producing augmented T-junction filtering in some instances. However, the robust filtering found in experimental studies may be produced by cellular mechanisms which occur over the span of several seconds, and therefore were not accounted for in our present study. Longer time-scale, likely calcium-dependent (45), mechanisms which regulate C-neuron excitability may be predominantly responsible for the robust augmentation of T-junction filtering during DRGS observed in preclinical studies (17). For example, region-specific calcium influx in the T-junction produced by suprathreshold DRGS may activate calcium-activated proteins such as calmodulin (74). Calmodulin regulates the currents produced by Nav1.8 (75), and may enhance steady-state inactivation of Nav1.8 channels (76). It is possible that long-term DRGS-induced calcium influx could modulate Nav1.8 currents, which may in turn make a cell likely to exhibit augmented T-junction filtering (Figures 9B, 10C). Data describing such phenomena in human DRG tissue would be critical to exploring the robustness of this effect in human C-neurons.

We believe that it is unlikely DRGS provides pain relief through a single mechanism. Conventional neurostimulation theory (20), previous computational studies of clinical DRGS (11, 12), and preclinical studies of DRGS in rodents (17) all suggest that large-diameter myelinated neurons are activated at lower thresholds than small-diameter non-myelinated neurons. Therefore, we believe that driving feed-forward pain-gating circuitry in the spinal cord *via* direct activation of Aβ-neurons is likely a key therapeutic mechanism of DRGS. However, direct activation of Aβ-neurons *via* DRGS may have indirect effects on C-neurons.

### Potential GABAergic effects during DRGS

Recent preclinical work demonstrated functional GABAergic signaling within the DRG, and that activation of DRG neurons which respond to GABA reduces nocifensive behaviors in rodents (52). Crucially, rodent DRG neurons which express 200 kDa neurofilament (i.e., large-diameter myelinated afferents, such as Aβ-neurons) possess the cellular machinery necessary to synthesize and package GABA into synaptic vesicles (52). Therefore, it is possible that DRGS directly activates Aβ-neurons, causing GABA release within the DRG which then modulates the activity of nearby C-neurons.

We simulated how GABA may affect the propagation of APs into the spinal axon of C-neurons. We first applied DRGS with a stimulus pulse amplitude of 1 mA (Figure 10A), an approximate upper bound for stimulus pulse amplitudes used clinically and likely sufficient to activate Aβ-neurons but not C-neurons (11, 23). Many (86.2%) model C-neurons did not change the number of spikes entering their spinal axons during 1 mA DRGS when including a somatic GABA conductance compared to without somatic GABA. A few model C-neurons (0.3%) reduced the number of spikes entering their spinal axons, while a small population of model C-neurons (13.5%) increased the number of APs entering their spinal axons when including a somatic GABA conductance. It is possible that GABA-evoked depolarizations spread electrotonically from the soma to the T-junction, facilitating the propagation of APs through the T-junction that would otherwise have failed. However, at pulse amplitudes used clinically, DRGS might not be causing significant effects on C-neurons through GABAergic mechanisms.

We then increased the DRGS pulse amplitude to a level sufficient to generate APs in C-neurons (i.e., suprathreshold DRGS). In response to suprathreshold DRGS (Figure 10B), including a somatic GABA conductance caused more C-neurons to decrease the number of spikes entering their spinal axons (37.3% of models) than models increasing or not changing the number of spikes (33.5% and 29.2%, respectively). Interestingly, models which experienced a decrease in APs propagating into their spinal axons had significantly greater maximal Nav1.8 and M-current conductances than models which experienced an increase in spinal axon APs (Figures 10C,D). These results suggest that direct activation of C-neurons coupled with somatic GABA currents may be sufficient for augmenting T-junction filtering of orthodromically propagating APs. However, somatic GABA currents may produce varying effects on C-neurons depending on their spatiotemporal ion channel expression profiles, and it is still unclear if C-neurons are being activated during clinical DRGS. Continued study of GABAergic signaling within DRG will be crucial to refining our understanding of how DRGS may engage such mechanisms.

### Modeling the response of populations of biophysically distinct neurons to electrical stimulation

We modeled the effects of therapeutic electrical stimulation on a population of biophysically distinct neurons, rather than a model with a single set of maximal ion channel conductances (11, 12, 16). We found that for a given stimulus pulse programming (e.g., pulse frequency, pulse duration), activation thresholds for a C-neuron at a single location in the DRG could vary by several mA depending on the maximal ion channel conductances used to parametrize the model (Figure 7). In general, this result suggests that activation thresholds for a given cell type may vary significantly depending on their unique spatiotemporal profiles of ion channel expression. We previously showed that two classes of Aδ-neuron models, which were morphologically identical but expressed different voltage-gated sodium channels, had different activation thresholds and activated to different extents by clinical DRGS (12). These findings suggest that the specific ion channel isoforms present in each cell type, and the relative expression of each ion channel in an individual cell of that type, may significantly affect its response to extracellular electrical stimulation. Understanding the variability between and within neuron populations may help improve the design of stimulation paradigms targeting these populations.

A key benefit of this population-modeling approach is that we can examine how each parameter used to construct a model may affect a metric of interest. For example, we found a negative correlation between maximal Nav1.8 conductance and activation threshold (Figures 7A,B), and a positive correlation between maximal M-current (Figure 7C) and SK (Figure 7D) conductance for three-and five-pulse activation thresholds. This result may suggest that fast-acting voltage-gated sodium channels responsible for the upstroke of the AP, such as Nav1.8 and Nav1.6 (77), may be a key determinant in setting a neuron's activation threshold to extracellular electrical stimulation. Furthermore, slower outward currents (e.g., SK), may influence a neuron's ability to respond to tonic electrical stimulation. Pharmacologically manipulating the function of one or more of these channels while simultaneously applying electrical stimulation may allow for the targeted control of a cell's ability to respond to a stimulus pulse. A dual pharmacologic and electrical approach may allow for greater neural selectivity than electrical stimulation alone if side effects can be managed adequately (78, 79).

A final benefit of this population-modeling approach is that it allows us to test hypotheses and the influences of design parameters on many biophysically plausible neuron models rather than on only a single model. Testing hypotheses on many models may reveal differential effects on neuron models depending on their specific biophysical parameters (25, 80). Similarly, this approach can simulate how novel neurotechnologies may engage with different neurons in a given population, bolstering the computer aided design process. Methods that account for how inherent biological variability can influence the cellular response to a potential therapy may improve efficacy of novel therapies.

### Limitations

We parametrized and validated our models using the best available published data. This approach necessitates building and validating models using data from several species because there are few studies describing such properties in human DRG. We were interested in how the electric fields generated by clinical DRGS systems modulate neural activity. Anatomical and technical factors, such as the position of highly resistive bone relative to the electrode array, can significantly affect thresholds in response to extracellular electrical stimulation (81). Therefore, we constructed the anatomy of the FEM model using anatomical data from human cadaver and imaging studies. However, there are few studies describing the properties of human DRG neurons, requiring us to parametrize and validate our C-neuron models on data from multiple species (e.g., rat, cat). Future studies should utilize human tissue to describe the morphological and electrophysiological characteristics (e.g., spatiotemporal ion channel expression profiles) of DRG neurons. Such data would greatly assist future studies modeling the neural response to clinical DRGS.

In this study, we parametrized our population of C-neuron models to reproduce experimentally measured electrophysiology characteristics (e.g., AP amplitude, AP rise time). Due to constraints on computational resources and the total simulation time needed to run the algorithm several times, we were unable to run a following frequency simulation for every iteration of the MCMC algorithm. Including a metric, such as following frequency, during the parametrization process may have yielded C-neuron models with more robust T-junction filtering. However, if voltage-gated ion channels play a key role in producing T-junction filtering, we believe that reproducing several electrophysiological characteristics of C-neurons is sufficient for a preliminary investigation into these phenomena. We also excluded axonal conductances from the population parametrization processes due to similar constraints on computational resources. However, ion channel types and densities present in the axons of DRG neurons have not been rigorously studied. Therefore, we believe the approach presented in this work serves as an excellent initial investigation into the impact of axonal slow ionic currents on AP propagation through the T-junction of C-neurons.

Another limitation to our study is that we modeled the temporal dynamics of GABA receptor activation as previously described in a model studying the effects of spinal cord stimulation on dorsal horn networks (53). Preliminary studies have examined which GABA subunits are present in DRG neurons (82). However, to the best of our knowledge, the dynamics of GABA receptor activation in DRG have not yet been quantified. A second key limitation is that we only modeled high-voltage activated L-type calcium channels in our model C-neurons. Low-voltage activated calcium channels, such as T-type calcium channels, have been shown to be upregulated in some chronic pain models (83). Because GABA depolarizes DRG neurons, low-voltage activated calcium channels may serve to augment the effects of GABAergic activation in DRG neurons, or trigger calcium-dependent mechanisms within the cell without producing a full AP. Despite these limitations, we believe our data represent a preliminary investigation into the effects of putative GABA release during DRGS, and highlight how inter-neuronal variability (e.g., maximal sodium conductance) may contribute to the population-level effects of GABA on the output of C-neurons to the spinal cord. Continued study of GABAergic signaling, particularly related to the role of satellite glial cells in GABA sequestration and release (84), will be crucial to determining the role of GABA in the analgesic mechanisms of DRGS.

## Conclusion

DRGS is an effective therapy for managing chronic pain that is refractory to conventional treatment strategies. However, not all patients receive sufficient pain relief from DRGS, and an incomplete understanding of its therapeutic mechanisms prevent us from optimizing the efficacy of this therapy. Therefore, in this study, we utilized a computational modeling approach to further investigate the neuromodulatory effects of DRGS. We implemented a field-cable modeling approach with a population of multi-compartment C-neuron models to investigate the hypothesis that DRGS augments the filtering of painful APs at the T-junction of nonmyelinated C-neurons. This population modeling approach highlighted the influence of several biophysical parameters in setting a neuron's response to extracellular electrical stimulation. In general, our models suggest that at clinical stimulation amplitudes, DRGS likely does not directly activate C-neurons nor does it robustly augment T-junction filtering. These findings contrast with available preclinical data, and several open questions remain regarding the influence of preclinical experimental design (e.g., electrode contact size) and the direct activation of nonmyelinated neurons by extracellular stimulation. The results of our study suggest that somatic GABA currents activated directly or indirectly by the DRGS pulse may produce diverse effects on orthodromic AP propagation in C-neurons. Going forward, close collaboration between computational and preclinical studies of clinical neurostimulation technologies, such as DRGS, will be crucial to improving our mechanistic understandings of these therapies and patient outcomes.

## Data Availability

The original contributions presented in the study are included in the article, further inquiries can be directed to the corresponding author.
